# A case report of single umbilical artery combined with fetal bladder exstrophy in singleton pregnancy and related literature review

**DOI:** 10.1186/s12884-024-06318-0

**Published:** 2024-02-09

**Authors:** Jun Zhan, Fenglin Jia, Qianqian Gao, Xue Xiao

**Affiliations:** 1 Department of Obstetrics and Gynecology, West China Second University Hospital, Sichuan University, Chengdu, China; 2 Key Laboratory of Birth Defects and Related Diseases of Women and Children (Sichuan University), Ministry of Education, No. 20 Ren Min Nan Road, Chengdu, Sichuan 610041 China; 3 Department of Radiology, West China Second University Hospital, Sichuan University, Chengdu, China; 4 Department of Ultrasound, West China Second University Hospital, Sichuan University, Chengdu, China

**Keywords:** Single umbilical artery, Bladder exstrophy, Fetal ultrasound, Fetal MRI, Cloacal exstrophy

## Abstract

**Background:**

According to prenatal ultrasonographic studies, single umbilical artery may be present alone or in association with other fetal abnormalities. So far, the exact pathogenesis of bladder exstrophy is unclear. Some scholars believe that bladder exstrophy and cloacal exstrophy should be regarded as a disease spectrum to explore their pathogenesis. If bladder exstrophy and cloacal exstrophy are regarded as the same disease spectrum, then we can speculate that the single umbilical artery should have the probability of being accompanied by bladder exstrophy at the same time.

**Case presentation:**

For the first time, we report a rare case of fetal bladder exstrophy with single umbilical artery in single pregnancy. This patient underwent targeted color Doppler ultrasound at 26 weeks of pregnancy which first suspected bladder exstrophy with single umbilical artery and fetal MRI for diagnosis at 38 + 3 weeks of pregnancy which confirmed the suspicion. After the diagnosis was confirmed, the patient was scheduled for a multidisciplinary discussion. Ultimately the patient opted for induced fetal demise at 38 + 5 weeks of pregnancy and the physical appearance of the fetal demise affirmed previous ultrasound and MRI examination results.

**Conclusions:**

Our report is the first finding of single umbilical artery combined with bladder exstrophy in a singleton pregnancy. Accordingly, our case enhances the evidence that cloacal exstrophy and bladder exstrophy should be treated as the same disease spectrum. In addition, we conducted a literature review on the diagnostic progress of single umbilical artery combined with bladder exstrophy, hoping to provide useful references for the diagnosis of this disease.

## Introduction

A normal umbilical cord has one umbilical vein and two umbilical arteries, and only one umbilical vein and one umbilical artery is called a single umbilical artery. The incidence of single umbilical artery is 1/500-1/50, and single umbilical artery is the most common umbilical cord dysplasia [1]. However, the mechanism of occurrence of single umbilical artery is not yet fully understood. Also, single umbilical artery can combine with various developmental disorders of organs and systems, such as cardiovascular malformations, genitourinary abnormalities, skeletal muscle deformations, gastrointestinal dysmorphias, brain abnormalities [2, 3]. Presently, the mechanism of abnormal fetal development combined with single umbilical artery is still inconclusive, but there are two current mainstream theories which are the theory of “vitelline vascular steal” [4] and the theory of “disturbance of embryo caudal development” [5].

Bladder exstrophy (hereinafter referred to as BE) is effectively short for bladder exstrophy-epispadias compound deformity, which is mainly characterized by the exposure to bladder mucosa. It is a rare and complex congenital malformation of the urogenital system. Main features of this condition include the absence of anterior bladder wall by different sizes, eversion of the posterior bladder mucosa, exposure to ureter and urethra and direct fusion of the posterior bladder wall with the lower abdominal wall skin [6]. The morbidity rate of BE is roughly 1/10,000-1/50,000, and the incidence ratio is proximately 1.5/1–5/1 as male to female on the basis of biological gender [7, 8]. Presently, the exact pathogenesis of bladder exstrophy is unclear. The mainstream view is that mesenchymal cells fail to migrate between the ectoderm of the abdomen and the cloaca during the fourth week of gestation, resulting to a series of abnormalities such as eversion of bladder on the abdominal surface, inferiorly displaced umbilicus, divergence of pubis, and abnormal external genitalia [9, 10].

Currently, some scholars believe that BE and cloacal exstrophy (hereinafter referred to as CE) should be regarded as a disease spectrum, and they believe that these two diseases are both manifestations of interference in early embryonic development [11–13]. It has been reported that the single umbilical artery can be associated with CE, and the bladder is originated from cloaca. Thus, if BE and CE are regarded as the same disease spectrum, then it should be inferred that the single umbilical artery can be accompanied by BE at the same time. So far, there are currently no reports of single umbilical artery accompanied by BE in single pregnancy. Thereby, for the first time, we report a rare case of fetal bladder exstrophy with single umbilical artery that was initially misdiagnosed as omphalocele by a primary hospital at 24 weeks of gestation, with the infant’s parents both Tibetans in China. This case adds to the evidence that CE and BE are the same disease spectrum. In addition, we conducted a literature review on the diagnostic progress of single umbilical artery combined with bladder exstrophy, hoping to provide useful references for the diagnosis of this disease.

## Case presentation

**Fig. 1:**
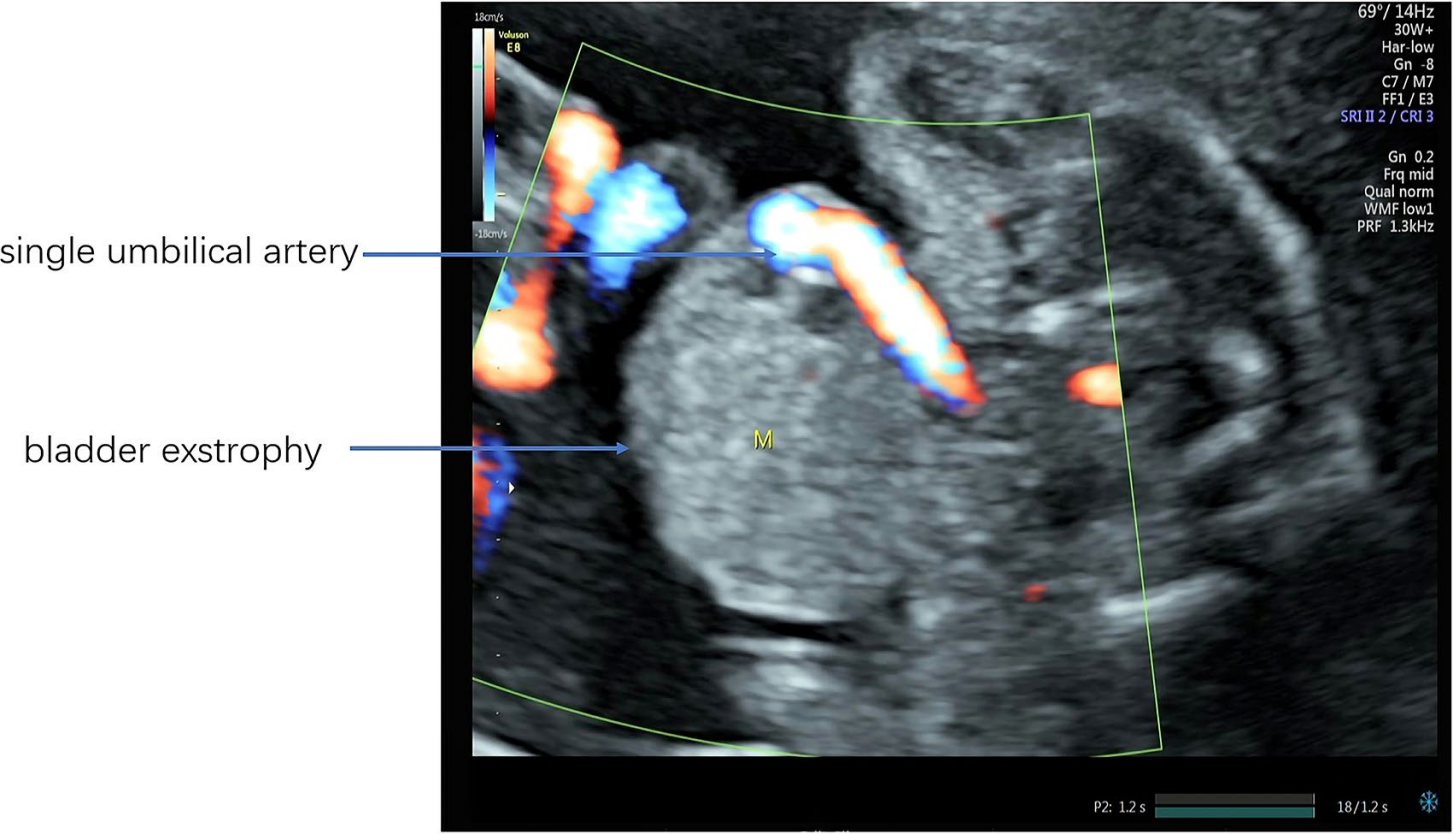
Transabdominal power Doppler image of the transverse section of the fetal bladder. The use of power Doppler ultrasound was to detect the level of the fetal bladder through the abdomen; there was no sign of bladder display in transverse section of fetal bladder plane; an isoechoic protrusion with a size of 1.82 cm × 1.58 cm was seen below the umbilical cord; only two blood vessels were seen in the umbilical cord

**Fig. 2:**
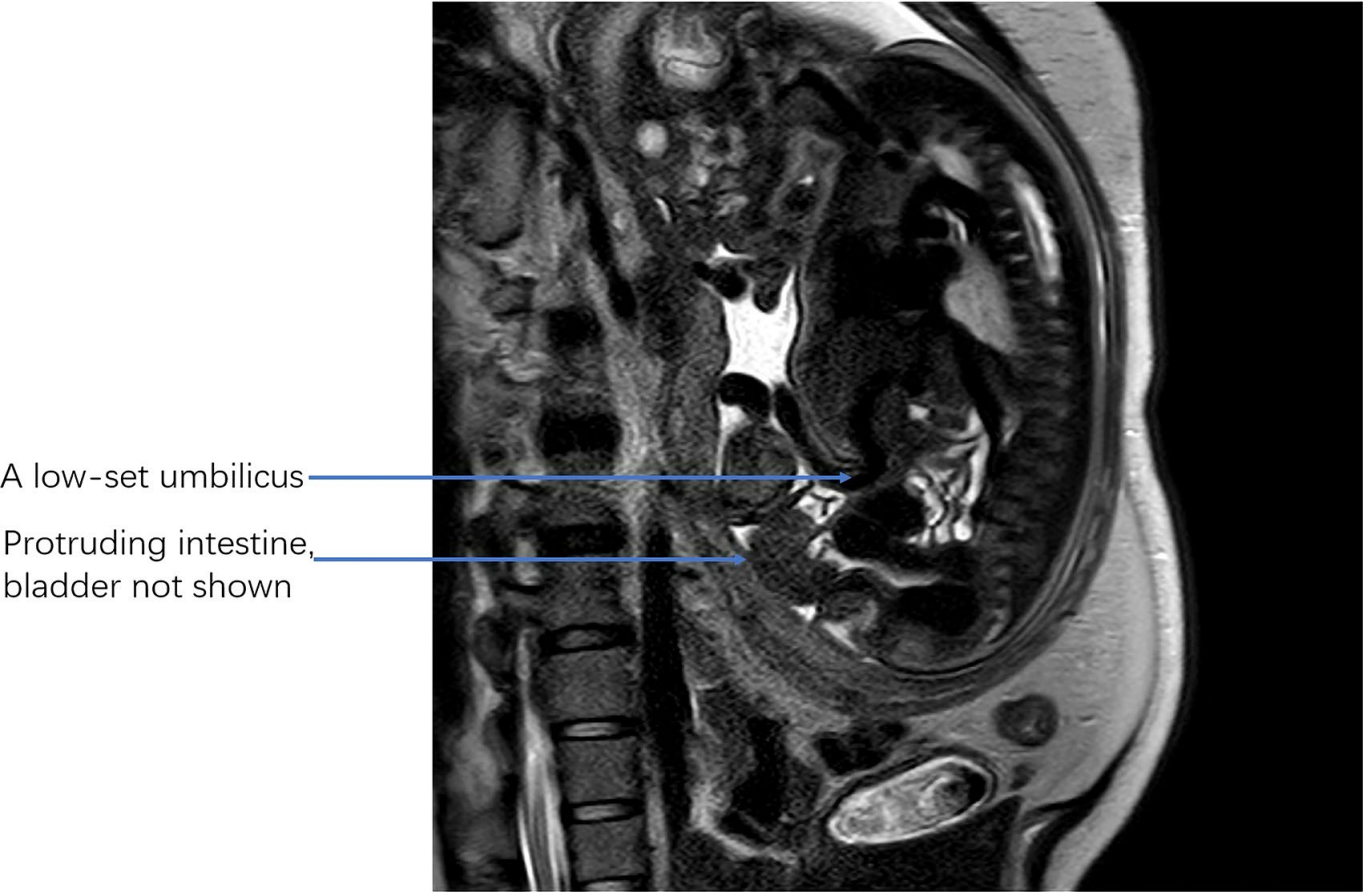
Fetal sagittal MRI T2WI image. Fetal sagittal MRI T2WI image showed that there was no high signal of bladder in the abdominal cavity; discontinuous abdominal wall and local protruding mixed signal shadows were detected; normal positions of the rectum and conus medullaris were indicated

**Fig. 3:**
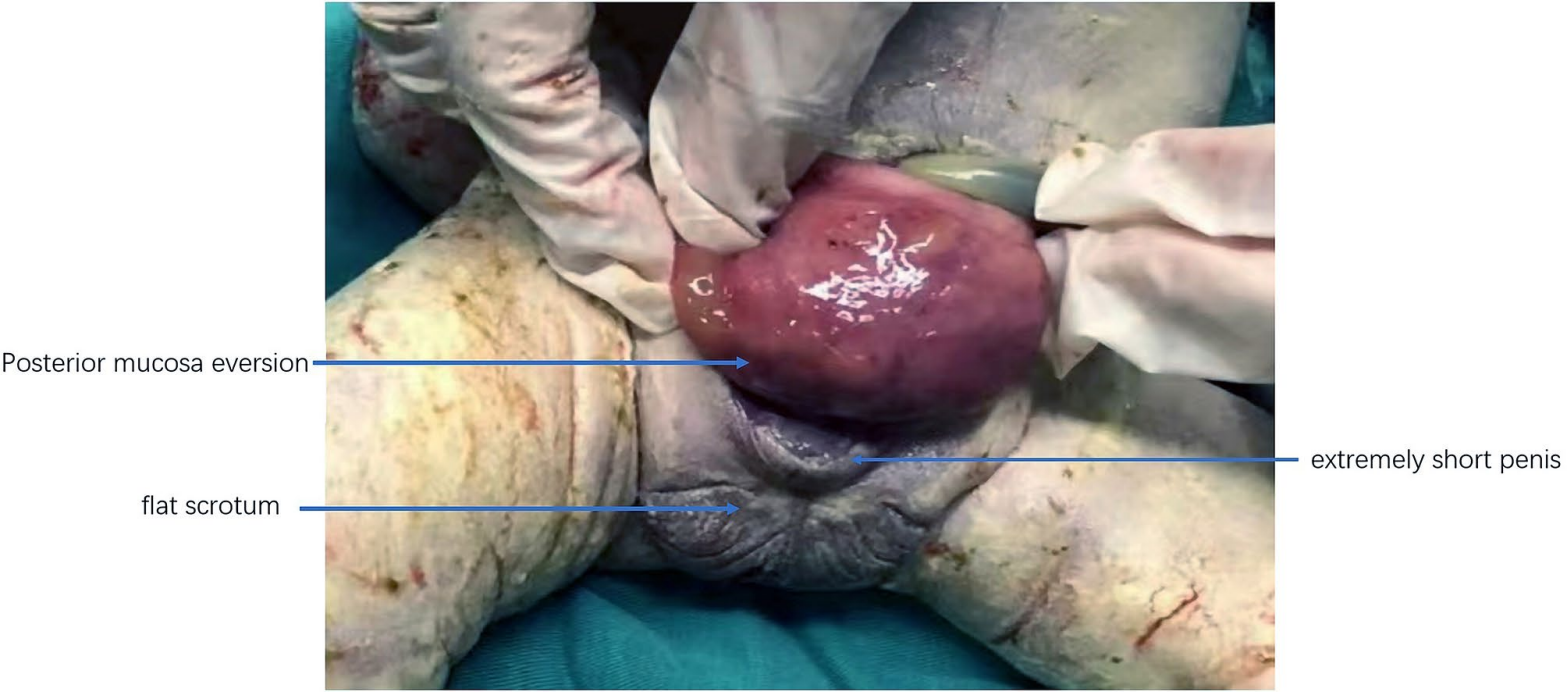
Macroscopic appearance of the fetus’s exstrophy of bladder and external genitalia. The macroscopic appearance of the infant showed a male dead infant with an abdominal wall defect of approximately 2 cm x3 cm below the insertion point of the umbilical cord. The infant had a posterior bladder mucosa eversion which was about 4 cm x 4 cm x 3 cm in size, a discontinuous symphysis pubis with proximate space of 2 cm, an extremely short penis, and a flat scrotum

This patient is a 35-year-old female with both her husband and herself Tibetans. She claimed no intermarriage and no medical history. Written informed consent was obtained from the patient for publication of this case report and any accompanying images. Pregnancy history showed G2P0 + 1 with spontaneous conception this time. She was diagnosed with hypothyroidism during the first trimester and was treated with 25 µg levothyroxine tablets which are taken orally every day. The color Doppler ultrasound examined in the first trimester showed no abnormalities, and patient claimed no history of exposure to toxic substances or radioactive substances. Non-Invasive Prenatal Testing (NIPT) results for the fetus indicated low risk. Furthermore, after several inquiries about medical history, the patient claimed no history of prior-conception diabetes. However, considering her overweight, she took an oral glucose tolerance test at 18 weeks of gestation and her fasting blood glucose was monitored to be normal with an 2-hour postprandial blood glucose exceeding 8.5 mmol/l which is less than 11.0 mmol/l. Therefore, the patient should be diagnosed as gestational diabetes mellitus rather than pregestational diabetes mellitus which at least requires one factor of the 2-hour postprandial blood glucose exceeding 11.0 mmol/l. At 24 weeks of pregnancy, she took a fetal 3D color Doppler ultrasound examination at a primary hospital and the results showed possible suspicions of omphalocele, rotarian placenta, single umbilical artery and fetal ventricular septal defect. Thus, she was referred to our hospital and received a targeted color Doppler ultrasound. There is no sign of bladder display (showed in Fig. 1), the insertion position of the abdominal wall of the umbilical cord is low, an isoechoic protrusion with a size of 1.82 cm × 1.58 cm is seen below the umbilical cord, the pubic symphysis is widened by about 2 cm, only two blood vessels are seen in the umbilical cord, the fetal heart ventricular septal defect is 2 mm and the depth of amniotic fluid is 8.2 cm. She then accepted amniocentesis for fetal chromosomal microarray analysis at 25 + 4 weeks of pregnancy, which showed no abnormal fetal karyotype. She was 28 weeks pregnant when she received this result. Then, she consulted with pediatric surgeons and physicians from pediatric cardiovascular department in our hospital respectively and both departments informed her to continue pregnancy and seek treatment immediately after the infant’s birth. Afterwards, she failed to go through regular obstetric examinations and was admitted to the hospital for elective cesarean section at 38 + 3 weeks of pregnancy. The physiological gender of the fetus is male according to fetal chromosomal microarray analysis, yet the ultrasound examination cannot accurately display the exact external reproductive organs. Furthermore, the physiological gender of the fetus is highly related to the prognosis considering that male’s reproductive system’s reconstruction surgery would be much more complicated and costly than that of female’s, which may place undue burdens upon patient’s financial situations and future life quality. In addition, a detailed explanation was given to the patient and her spouse on the necessity of conducting an MRI examination. The patient fully understood and consented the MRI examination which contained a comprehensive evaluation of the fetus, especially the external genitalia (showed in Fig. 2). The results showed that the axial view of T2WI indicated bladder exstrophy, and there was no high signal of bladder in the abdominal cavity; sagittal view showed the high signal of low insertion of the umbilical cord; the external genitalia were unclear; both sagittal and axial T2WI showed discontinuous abdominal wall and local protruding mixed signal shadows; sagittal T1WI and T2WI showed normal positions of the rectum and conus medullaris; no obvious abnormalities in the shape and size of both kidneys; and no dilation of bilateral ureters. No abnormality was found in other tissues and organs. We arranged a multidisciplinary team (MDT) discussion for her including a genetic counselor, ultrasound physician, radiologist, pediatric urologist and obstetrician. It was clearly explained to her and her husband that the fetus was diagnosed with bladder exstrophy accompanied by abnormal external genitalia, and the physiological gender of the fetus is male. Further examinations were necessary after birth, and multiple surgical treatments were required. The surgery was complicated and difficult, and the prognosis was uncertain. Also, there may be risks of urinary tract obstruction and infection during childhood, uncontrollable urination, large scars on the abdominal wall and inability to urinate spontaneously which may affect future sexual activities and quality of life at high costs. The patient and her family eventually decided to induce fetal demise. This patient underwent amniocentesis and injection of ethacridine for induced labor. Two days later, a male dead infant was delivered (showed in Fig. 3). The macroscopic appearance of the infant showed an abdominal wall defect of about 2 × 3 cm below the insertion point of the umbilical cord. Posterior mucosa eversion, about 4 × 4 × 3 cm in size, discontinuous symphysis pubis with proximate space of 2 cm, extremely short penis, flat scrotum, testicles are palpated in it, and there are only two blood vessels in the umbilical cord. On the third day after delivery, the patient was discharged without any discomfort. The patient and their family expressed satisfaction with the treatment plan.

## Discussion

### Discussion on the pathogenesis of single umbilical artery combined with bladder exstrophy

#### Single umbilical artery may contribute to fetal malformations

Single umbilical artery is the most common umbilical cord dysplasia. The mechanism of occurrence of single umbilical artery is not fully understood yet. There are generally three theories: one is that there is only one umbilical artery when the embryo begins to develop; the other theory is that there are two umbilical arteries when the embryo begins to develop, but one of them gradually shrinks or becomes atresia in later development; and the third one is the persistence of the primitive allantoic artery of the body stalk. However, some scholars have proposed another possible mechanism of single umbilical artery: the allantoin-derived umbilical artery stops developing, or becomes atrophy or atresia after forming a normal umbilical artery, or the degeneration of yolk-derived vasculature fails [14]. Moreover, some scholars believe that the occurrence of single umbilical artery is not caused by a single high-risk factor, but may be related to genetics, environment or other factors [15]. Single umbilical artery can combine with malformation of multiple organs and systems in the fetus, and Staribratova [2] found that it was associated with an increased incidence of hollow organ atresia, renal abnormalities, limb reduction defects, and spontaneous abortion. Friebe-Hoffmann [3] conducted a retrospective analysis of 1169 singleton pregnancies prenatally diagnosed as single umbilical artery from 1997 to 2014. They found that 989 (84.6%) fetuses showed a single umbilical artery without any combinations. And 180 cases (15.4%) fetuses not only had a single umbilical artery, these cases also had structural abnormalities or chromosomal abnormalities. The structural abnormalities were mainly: 9.0% cardiovascular abnormalities, 3.5% genitourinary abnormalities, 2.9% skeletal muscle abnormalities, 3.0% gastrointestinal abnormalities, 2.1% brain abnormalities, and 2.1% fetal chromosomal abnormalities.

The current mechanism of abnormal fetal development combined with single umbilical artery is mainly the theory of “vitelline vascular steal” [4] and “disturbance of embryo caudal development” [5].

The “vitelline vascular steal” theory holds that a thick malformed vessel is derived from the vitelline artery which originates from the high abdominal aorta and performs the function of the umbilical artery. This artery transports blood from the umbilical cord to the placenta, while the abdominal aorta is often small and has no branches. Thus, the thick and deformed blood vessels “steal” a large amount of blood from the abdominal aorta into the placenta, causing its origin to be far from the abdominal aorta which leads the blood to be significantly reduced, and it contributes a serious lack of blood supply to various structures of the fetus. These in turn may lead to severe deformities of spine, lower limbs, kidneys, lower gastrointestinal tract, urogenital tract, and reproductive organs. Another study found that a single umbilical artery has a clear and definite relationship with the occurrence of caudal defect syndrome (such as Sirenomelia which is also called the mermaid syndrome). This discovery supports the theory of “vitelline vascular steal” which leads to abnormal fetal development, and the degree of blood loss and the timing of the embryonic developmental process may determine the exact type of defect [4].

Another theory of “disturbance of embryo caudal development” suggests that the umbilical artery is the largest caudal branch to the embryonic dorsal artery. If the primordium of the umbilical artery is not formed, the development of the dorsal caudal artery of the embryo will be abnormal. In addition, the development of embryos supplied by the caudal artery will be affected, resulting in deformities of the corresponding organ structures, which can lead to developmental deformities in three aspects: (1) the blood supply of the lower part of the early embryo is affected, resulting in abnormalities in the cloaca, urogenital tract, stomach and other organs or resulting in developmental malformations of the gut, central nervous system, and lower extremities; (2) there may be disturbances in the formation of the anterior abdominal wall below the umbilical cord; (3) embryonic hemodynamics is altered, resulting in cardiovascular malformations and possible development of defects toward the embryonic head [5].

#### Possible mechanism of occurrence of bladder exstrophy

BE is effectively short for bladder exstrophy-epispadias compound deformity, which is mainly characterized by the exposure to bladder mucosa. It is a rare and complex congenital malformation of the urogenital system. Main features of this condition include the absence of anterior bladder wall by different sizes, eversion of the posterior bladder mucosa, exposure to ureter and urethra and direct fusion of the posterior bladder wall with the lower abdominal wall skin [6]. The morbidity rate of BE is roughly 1/10,000-1/50,000, and the incidence ratio is proximately 1.5/1–5/1 as male to female on the basis of biological gender [7, 8]. Presently, the exact pathogenesis of bladder exstrophy is unclear. The mainstream view is that mesenchymal cells fail to migrate between the ectoderm of the abdomen and the cloaca during the fourth week of gestation, resulting to a series of abnormalities such as eversion of bladder on the abdominal surface, inferiorly displaced umbilicus, divergence of pubis, and abnormal external genitalia [9, 10]. Another hypothesis proposed by Satish is that pubic symphysis diastasis triggers a hammock status of levator ani muscles which forces the pelvic organs to move forward, resulting in the stretching, thinning and rupture of bladder and anterior abdominal wall. This aforementioned process is related to the formation of BE [16]. The etiology of bladder exstrophy is unknown but may be related to genetic, environmental and other factors. Most patients have normal chromosomal phenotypes, but Catharina et al. pointed out that rare genetic copy number variations may trigger BE [17]. In this case, the patient was elderly parturient and the ultrasound examination suspected several other abnormalities rather than bladder exstrophy, including single umbilical artery and fetal ventricular septal defect. Therefore, a fetal chromosome test was undergone, but the results came back negative.

#### Discussion on the relationship of bladder exstrophy and cloacal exstrophy

At present, some scholars believe that bladder exstrophy and cloacal exstrophy should be regarded as a disease spectrum for clinical purpose. These scholars believe that both cloacal exstrophy and bladder exstrophy are abnormalities of early embryonic development. They suggest that damage that occurs early in embryonic development leads to cloacal exstrophy. “Hits” occurs later in embryogenesis, the period after the urogenital septum has reached the cloacal membrane, leading to bladder exstrophy [11–13]. These malformations may also result from environmental or genetic abnormalities that impede the formation of the last somites of caudal, thereby preventing normal urorectal septum and allantois development [18].

#### Single umbilical artery may combine with bladder exstrophy

Several cases of single umbilical artery combined with cloacal exstrophy have been reported previously [18–20]. Therefore, if BE and CE are regarded as the same disease spectrum, then we can speculate that the single umbilical artery should have the probability of being accompanied by BE at the same time. However, previous cases have only reported that single umbilical artery and CE can be accompanied simultaneously, and so far, there is no report of single umbilical artery combined with BE in singleton pregnancy. Martínez-Frías, Bermejo, Rodríguez-Pinilla and Frías [21] investigated 1,601,860 live births and discovered that 8 cases had cloacal exstrophy, and about 37.5% of them were associated with single umbilical artery. However, no single umbilical artery was found to be associated with BE before. Therefore, our case which BE is accompanied by single umbilical artery strengthens the evidence that CE and BE are the same disease spectrum. And our case provides a new clinical evidence for the above theory, hoping to provide a new perspective for related theories.

### How to diagnose single umbilical artery combined with bladder exstrophy

When the fetus has a single umbilical artery, two-dimensional ultrasound will show that it is in the shape of two parallel circles, and the long-axis section will show that the spiral is sparse. In addition, color Doppler ultrasonography will show that the umbilical artery and umbilical vein are running in parallel, and in the transverse section at the level of the fetal bladder, only the umbilical artery blood flow signal will be seen on the bladder side (Fig. 1).

When the fetus is associated with BE, the ultrasound findings are as follows: (1) no bladder can be seen in the pelvic cavity of the fetus; (2) there is a protruding mass in the abdominal wall of the lower abdomen; (3) the kidneys are normal and the amniotic fluid volume is normal; (4) the physiological gender of the fetus is difficult to determine. Details are as follows:

Absence of bladder indication on prenatal ultrasonography is the first clue to the diagnosis of bladder exstrophy [22]. Normally, the fetal bladder can be fully displayed on ultrasound at 14 weeks of gestation, and the bladder is filled and emptied every 30 to 40 min. However, the posterior wall of the bladder with diagnosis of BE is directly adjoined with the abdominal wall, and the ureter on the bladder wall is exposed. Urine is drained directly into the amniotic cavity, and there is no urine filling in the bladder [23]. Bladder exstrophy can be diagnosed if urine is directly jetted into the amniotic cavity during fetal examination. This approach can shorten examination time and avoid waiting for possible bladder filling or multiple targeted ultrasounds to confirm the diagnosis. The dynamic observation of the fetal bladder is quite important during the second trimester structural screening. Especially when there is no obvious abnormality in both kidneys of the fetus and the amniotic fluid volume is normal, the disease should be highly suspected when the bladder between the bilateral umbilical arteries is not filled continuously for 30–60 min [24]. However, the bladder can have an alternating indication of filling and emptying, especially in fetuses with normal kidneys and normal amniotic fluid volume, sonographers tend to misidentify absence of bladder as bladder emptying, thus bladder exploration is easy to be ignored in sonography examination. Prenatal ultrasound of fetal bladder exstrophy often shows that the bladder between the two umbilical arteries in the lower abdomen is not continuously visible. Therefore, several studies suggest measuring the bilateral umbilical arteries when the bladder is not visualized [25]. However, in some rare cases as our case here, there is only one umbilical artery making it nearly impossible to assist diagnosis by measuring the umbilical artery. In addition, the urachal cyst in the lower abdomen and the umbilical cords on both sides are similar to the normal bladder, and the true bladder exstrophy is easy to be missed in some rare cases [16]. Prenatal diagnosis of bladder exstrophy becomes more difficult when the bladder is not visible which is also accompanied by bilateral renal malformations and oligohydramnios. Fetal structural malformation screening is usually performed near the 24th week of gestation. When diagnosed as bladder exstrophy, the gestation often reaches the later stage. In some special cases, fetal karyotypes are even required. It is often in the third trimester when the results of fetal karyotypes are received. Because of the late stage of gestation and related legal issues, the best time to terminate the pregnancy has passed. Therefore, methods for early diagnosis of this disease are urgently needed. Fishel-Bartal [22] discovered that the umbilical cord insertion–to–genital tubercle length of fetuses with bladder exstrophy was below the fifth percentile of the general population. This measurement can be used as a complementary objective ultrasound parameter in prenatal assessment and used for the diagnosis of suspected cases in early pregnancy. Under normal circumstances, an equilateral triangle consisting of the aorta, umbilical artery, and umbilical vein can be seen under color Doppler ultrasound, and Kavita Aneja [26] discovered that when the fetal bladder is everted, this equilateral triangle can deform. Thus, it is a finding that helps detect BE in early pregnancy.

A low-set umbilicus is another unique characteristic of bladder exstrophy, but currently neither ultrasound nor MRI has an objective reference point for evaluating the location of the fetal umbilical cord entry [27]. The judgment of the location of the umbilical cord mainly depends on the experience of the sonographer. Gilboa [28] suggested that the umbilical cord insertion-to-genital tubercle length of the fetus at 12 to 18 weeks of gestation was below the 5th percentile of the normal range, indicating a diagnosis of bladder exstrophy. Howbeit, it is difficult to obtain this section in fetuses over 17 weeks, which reduces the application value of this method in the diagnosis of BE.

A lower abdominal mass is another sign of BE, this mass is the bulging back wall of the bladder and bowel, mostly located below the entrance of the umbilical cord. Unfortunately, due to the occlusion of the umbilical cord set, the low intra-abdominal pressure, the non-protrusion of the everted bladder and the fetal position, the detection rate of the sub umbilical bulge mass is merely 28–47% [29]. In recent years, some scholars have also suggested that MRI is better at examining a lower abdominal mass than ultrasound does. When prenatal ultrasonography suspects BE but does not detect a lower abdominal mass, it is recommended to perform fetal MRI to assist the diagnosis. MRI can effectively identify ureteral and bladder wall abnormalities without possible interference by maternal body habitus, amniotic fluid volume and fetal position [30].

Frequently, BE is also accompanied by widened pubic rami. Prenatal ultrasound can determine the degree of pubic symphysis separation by measuring the distance of the fetal symphysis. Antomarchi [27] measured the pubic symphysis distance in 868 fetuses and found that this method was feasible in 71% of cases before 27 weeks of gestation. He then proposed that it can be used to distinguish almost any normal and pubic separation cases of age when the cut-off value is 1 cm, however, it is inapplicable in 60% of fetuses above 32 weeks of gestation.

BE is often associated with abnormal external genitalia, and the prognosis of male infants is worse than that of female infants. Prenatal ultrasound examination of fetal external genitalia can provide an important basis for the diagnosis of BE [31]. However, there are plenty types of external genital abnormalities. Thus, when prenatal ultrasound and MRI examinations are difficult to identify fetal gender, fetal chromosome examination can be recommended. In our case, the external genitalia were not clearly displayed on color Doppler ultrasound, and the physiological gender of the fetus is highly related to the prognosis. Therefore, we arranged fetal MRI for her, but the external genitalia were still not clearly displayed (Fig. 2). The fetus also had a ventricular septum and a single umbilical artery, so the patient underwent amniocentesis for fetal chromosome examination at 25 + 4 weeks of pregnancy. The chromosome result showed that it was male, and no other abnormal results were found. The patient and her family eventually chose to induce fetal demise because they were worried about problems related to the prognosis of the newborn, such as uncertain prognosis, discrimination, trauma and cost of multiple surgeries, as well as psychological trauma and sexual issues in adulthood.

## Differentiating diagnosis

BE is not difficult to diagnose when there is a typical indication of absence of bladder filling. BE should be differentially diagnosed apart from omphalocele, gastroschisis and cloacal exstrophy, patent urachal prolapse with bladder prolapse or patent urachal cyst with allantoic cyst [32–35]. The former two can show a normal bladder in the pelvis. However, when intestinal, anal atresia, spinal cord abnormalities and external genitalia are present at the same time, cloacal exstrophy should be suspected [36]. The latter two usually exist in isolation, and they often do not appear together with symphysis pubis separation or external genital malformation.

## Conclusion

To our knowledge, this case report is the very first one to discover that BE can occasionally be incorporated with single umbilical artery. Our case may supplement the evidence that BE and CE are the same disease spectrum. Furthermore, BE is a rather rare condition and may sometimes be associated with some soft fetal ultrasound markers or abnormalities excluding genitourinary. With the widespread popularity of prenatal diagnosis, such deformed fetuses can be diagnosed during the second trimester. Thus, we believe that arranging for an MDT to assist the diagnosis and treatment of this rare condition is beneficial to patients which can also save patients’ time for seeking multiple treatments thus avoiding inconsistent consultation results between departments. How to diagnose this disease promptly is still a clinically challenging task. We recommend performing a fetal ultrasonography between 14 and 20 weeks of gestation and paying attention to see if the bladder is visible, and pay attention to the fetal umbilical cord. In addition, target ultrasonography and/or MRI may be helpful for accurate recognition.

## Data Availability

The datasets used and/or analyzed during the current study are available from the corresponding author on reasonable request.

## Abbreviations

BE: Bladder exstrophy
CE: Cloacal exstrophy

## Declarations

### CARE guidelines compliance

This study adheres to all CARE guidelines and a completed CARE checklist file has been uploaded as a supporting file in the submission system.
